# Levels, timing, and etiology of stillbirths in Sylhet district of Bangladesh

**DOI:** 10.1186/1471-2393-11-25

**Published:** 2011-04-01

**Authors:** Abdullah H Baqui, Yoonjoung Choi, Emma K Williams, Shams E Arifeen, Ishtiaq Mannan, Gary L Darmstadt, Robert E Black

**Affiliations:** 1 Department of International Health, Bloomberg School of Public Health, Johns Hopkins University, Baltimore, MD, USA; 2 Public Health Sciences Division, ICDDR, B, Dhaka, Bangladesh; 3 Family Health Division, Global Health Program, Bill & Melinda Gates Foundation, Seattle, WA, USA

## Abstract

**Background:**

Lack of data is a critical barrier to addressing the problem of stillbirth in countries with the highest stillbirth burden. Our study objective was to estimate the levels, types, and causes of stillbirth in rural Sylhet district of Bangladesh.

**Methods:**

A complete pregnancy history was taken from all women (n = 39 998) who had pregnancy outcomes during 2003-2005 in the study area. Verbal autopsy data were obtained for all identified stillbirths during the period. We used pre-defined case definitions and computer programs to assign causes of stillbirth for selected causes containing specific signs and symptoms. Both non-hierarchical and hierarchical approaches were used to assign causes of stillbirths.

**Results:**

A total of 1748 stillbirths were recorded during 2003-2005 from 48,192 births (stillbirth rate: 36.3 per 1000 total births). About 60% and 40% of stillbirths were categorized as antepartum and intrapartum, respectively. Maternal conditions, including infections, hypertensive disorders, and anemia, contributed to about 29% of total antepartum stillbirths. About 50% of intrapartum stillbirths were attributed to obstetric complications. Maternal infections and hypertensive disorders contributed to another 11% of stillbirths. A cause could not be assigned in nearly half (49%) of stillbirths.

**Conclusion:**

The stillbirth rate is high in rural Bangladesh. Based on algorithmic approaches using verbal autopsy data, a substantial portion of stillbirths is attributable to maternal conditions and obstetric complications. Programs need to deliver community-level interventions to prevent and manage maternal complications, and to develop strategies to improve access to emergency obstetric care. Improvements in care to avert stillbirth can be accomplished in the context of existing maternal and child health programs. Methodological improvements in the measurement of stillbirths, especially causes of stillbirths, are also needed to better define the burden of stillbirths in low-resource settings.

## Background

Stillbirth has long been a large, yet mostly hidden burden of disease in the developing world [1-3]. Recent studies have drawn attention to the high global burden of an estimated 3.2 million annual stillbirths [2,4]. Most stillbirths could be prevented by improving access to quality prenatal and obstetric care that is standard in wealthy countries [2]. Global estimates suggest that about one-third of stillbirths occur during the intrapartum period [5]. However, a five-country prospective study of stillbirth found only 17% of stillbirths were macerated, suggesting a higher proportion were intrapartum [6]. Estimating the timing of stillbirth is often challenging in low resource settings,[2,7] and timing could not be determined in 46% of the stillbirths in an Egyptian study [7]. Nevertheless, some estimates suggest that up to 50% of stillbirths may be attributed to intrapartum conditions in settings with poor access to obstetric care [3]. The primary causes of stillbirth are thought to be prolonged and obstructed labor, hypertensive disorders of pregnancy and maternal infections [2,3].

Lack of data in countries with the highest stillbirth rates, due to poor vital registration systems and a low proportion of births or deaths in health facilities, is a critical barrier to addressing the problem of stillbirths [1,2,8,9]. Measurement of stillbirths in community settings is particularly challenging, and verbal autopsy studies have often been used to assign causes of under-five child and neonatal deaths in settings where most deaths occur outside of facilities or a physician's care [9-15]. This methodology has also been used recently to ascertain causes of stillbirth [7,16-18]. The main objectives of this paper were to estimate rates, types, and causes of stillbirths, using pregnancy history and verbal autopsy data from a large-scale survey conducted in rural Sylhet district of Bangladesh.

## Methods

### Study Population and Design

The study population came from the endline survey of the Projahnnmo-1 project, a cluster-randomized, controlled trial of a package of interventions to improve maternal and newborn health and survival. The trial was conducted in three rural sub-districts of Sylhet district, located in the northeastern part of the country [19]. A 34% reduction in neonatal mortality associated with the interventions was reported, and information on the design, implementation, and evaluation of the trial has been presented in detail elsewhere [19]. The baseline neonatal mortality rate was 48 per 1000 live births and the stillbirth rate was estimated at 32 per 1000 total births [20]. Health services are provided by the public sector, nongovernmental organizations, and a large formal and informal private sector. The closest emergency obstetric care facility is outside the study area at the Medical College Hospital in Sylhet city. The mean direct distance between the hospital and the 611 villages in the study area is 42.8 km (standard deviation 12.4, range [17.2-64.5]). Women in Sylhet division (a larger administrative area that Sylhet district belongs to) as a whole had a cesarean section rate of 4.7% [21].

### Data

The endline survey of the Projahnmo-1 project, conducted during January - June 2006, collected data on lifetime pregnancy history and demographic and household wealth information from all women in the study area who had a pregnancy outcome in the three previous calendar years (2003-2005) [19]. Pregnancy outcomes were classified as miscarriage, stillbirth, and live birth. Stillbirth was defined as delivery of a dead fetus with a gestational age of 7 months or more, and miscarriage was defined as the loss of a fetus of gestational age less than 7 months based on respondents' recall of first day of last menstrual period. For stillbirths, a follow-up question was asked to confirm that the baby never breathed or cried after birth.

For all stillbirths which occurred during 2003-2005, a verbal autopsy was conducted by separate interviewers who had at least 12 years of schooling and had received six days of training in verbal autopsy data collection. A total of 27 interviewers participated in data collection, and each collected information for about 65 stillbirths on average (standard deviation: 27, range [5]113). The verbal autopsy interview was conducted during April - September 2006. We used the revised World Health Organization standard neonatal verbal autopsy tool,[22] in which all information is based on interviewees' report. The tool distinguishes stillbirths and early neonatal deaths and includes both open-history narratives and closed-ended questions about signs and symptoms of illness leading to death [15,22].

### Measurement and Analysis

Stillbirth rate was calculated as the number of stillbirths per 1000 total births; the denominator included both stillbirths and live births. Stillbirths were categorized into two types based on information suggesting the timing of stillbirth relative to labor and delivery: antepartum and intrapartum. Stillbirths were classified as antepartum if the respondent reported "skin and tissue was pulpy (i.e., macerated body)" or "baby stopped moving before labor" (i.e., 'baby stopped moving during pregnancy' or 'baby did not move during the last few days before the birth').

Within each type of stillbirths, we used computer-based algorithms containing specific signs and symptoms (i.e., pre-defined expert algorithms) to assign causes of stillbirths, and allowed multiple causes for each stillbirth. We reviewed the recently suggested classification system for global estimates of causes of stillbirth[2] and assigned selected causes which could be measured using verbal autopsy data and that reflected maternal and fetal complications associated with potential underlying causes of stillbirth (Table 1) [16,23-26]. The five causes of antepartum stillbirths were congenital anomalies, maternal hemorrhage, maternal hypertensive disorders, maternal infections, and maternal severe anemia. For intrapartum stillbirths, we assigned five causes: congenital anomalies, intrapartum hypoxia, preterm labour, maternal hypertensive disorders, and maternal intrapartum infections. Maternal hypertensive disorders include pregnancy-induced hypertension, preeclampsia, and eclampsia. The case definition of maternal infections referred to a wide range of infections during pregnancy, including urinary and reproductive tract infections, sexually transmitted infections, and infection induced by or causing premature rupture of membranes [27]. Potential overlapping multiple causes were examined by cross-tabulation of all causes.

**Table 1:** Verbal autopsy case definitions for selected causes of antepartum and intrapartum stillbirths

Timing of stillbirth	Cause	Case definitions applied in verbal autopsy data
Antepartum ('skin and tissue was pulpy' ***or ***'baby stopped moving before labor')	**Congenital abnormality**	very small head/no brain at the time of birth; OR mass or defect on the back of the head or spine
	**Maternal hemorrhage**	vaginal bleeding during pregnancy
	**Maternal hypertensive disorders**	convulsion; OR hypertension during pregnancy diagnosed by a health worker*
	**Maternal infections**	fever during delivery; OR green/brown color or foul smelling amniotic fluid
	**Maternal severe anemia**	severe anemia during pregnancy†
Intrapartum (***Neither ***'skin and tissue was pulpy' ***nor ***'baby stopped moving before labor')	**Congenital abnormality**	very small head/no brain at the time of birth; OR mass or defect on the back of the head or spine
	**Intrapartum hypoxia**	breech presentation; hand/feet delivered first; cord delivered first; obstructed labor; prolonged labor, OR vaginal bleeding during pregnancy
	**Preterm labour**	Gestational age < 8 months
	**Maternal hypertensive disorders**	convulsion; OR hypertension during pregnancy diagnosed by a health worker*
	**Maternal intrapartum infections**	fever during delivery; OR green/brown color or foul smelling amniotic fluid

*Reported history of having the diagnosis by a health worker

†Reported, which may or may not be based on clinical diagnosis by health workers

**Figure 1:**
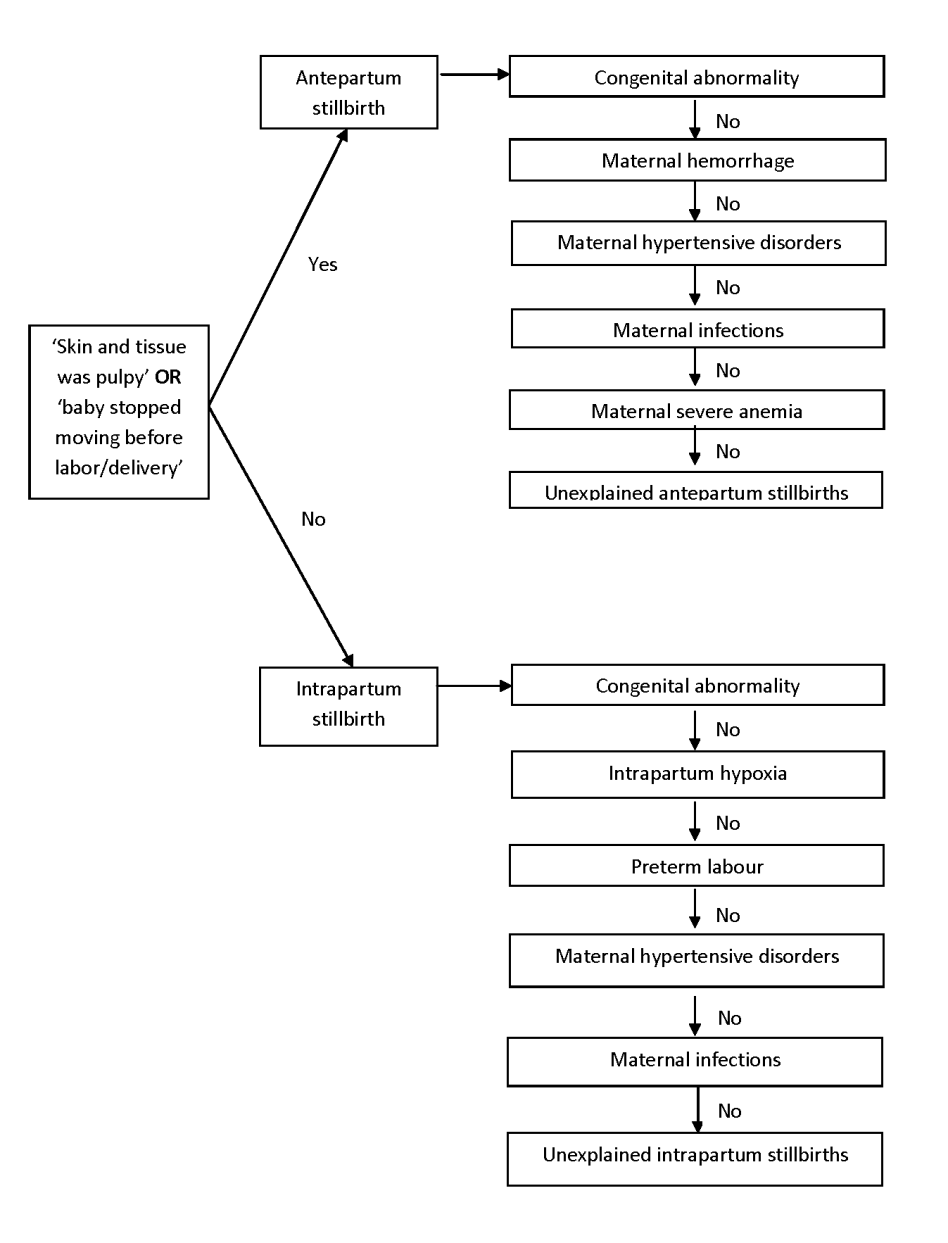
Classification of stillbirths by timing and hierarchical algorithm assigning primary causes

A non-hierarchical approach was used first to assign all possible causes among thosedescribed above, resulting in multiple causes for some stillbirths. Then, ahierarchical approach was used to assign a single primary cause of stillbirth, again using computer-based hierarchical algorithms. We reviewed the presumed physiological precedence of each cause's contribution to stillbirths and also considered probable accuracy of each case definition in our measurement, based on the exactness of signs and symptoms described in the verbal autopsy questionnaire. Cases with presumed high specificity were placed at a higher level in the hierarchy, although this study did not permit validation of case definitions. We adapted the hierarchies used in the Obaapa study[16] - one of the few which validated hierarchical algorithms using verbal autopsy data to ascertain a primary cause of stillbirths - with a few modifications (Figure 1).

Preliminary analyses suggested that stillbirth rates did not vary across study arms [19]. Thus, we used pooled data across study arms to provide estimates for the entire study area during 2003-2005. Analysis on timing and causes of stillbirths was limited to the stillbirths for which the mother was the main respondent for a completed verbal autopsy interview, in order to eliminate potential reporting errors on maternal complications by a third person [28]. We included both singleton and multiple births in analyses. STATA 10.0 statistical software (Stata Corporation, College Station, TX, USA) was used for the analysis.

The study was approved by the Institutional Review Board at the Johns Hopkins Bloomberg School of Public Health, and the Ethical Review Committee and the Research Review Committee at the International Center for Diarrhoeal Disease Research, Bangladesh. The study was registered at clinicaltrials.gov, No. 00198705.

## Results

A total of 48,192 births and 1,748 stillbirths were recorded during 2003 - 2005, resulting in a stillbirth rate of 36.3 per 1000 total births (95% CI: 34.6-38.0 per 1000 total births) (Table 2). Verbal autopsy interviews were completed for 1584 stillbirths (90.6%). The most common reason for incomplete interview was absence of any respondent (45%, 74/164). Background characteristics and recall period between pregnancy termination and verbal autopsy interview did not vary by interview status (Table 3). Among those who completed the interview, about 80% delivered at home, 20% were assisted by neither health personnel nor a traditional birth attendant, and the majority delivered vaginally without any assistance or only with manual assistance (Table 3). The main respondent was the mother in 98.1% (1554/1584) of the completed interviews. Among the 1554 stillbirths, 164 (10.6%) were reported by 80 women who had 2 or more stillbirths during the period through multiple births and/or repeated pregnancies. The mean recall period was 25 months (standard deviation: 11, range [3,44], n = 1554).

**Table 2:** Number of births and stillbirths by year

Year	Births	Stillbirths							
		
		Total	Rate (per 1000 total births)	Completed VA interview*	Complete VA interview with mother				
					
					Total	Antepartum		Intrapartum	
						n	(%)	n	(%)
2003	16,985	609	35.9	544	537	323	(60.1)	214	(39.9)
2004	15,392	563	36.6	510	492	302	(61.4)	190	(38.6)
2005	15,815	576	36.4	530	525	340	(64.8)	185	(35.2)
**Total**	**48,192**	**1,748**	36.3	**1,584**	**1,554**	**965**	(62.1)	**589**	(37.9)

VA: Verbal Autopsy

Distributions of stillbirths by timing did not vary by year (chi-square test p-value 0.278).

*interview was completed by any adult informants such as mothers, fathers, and other relatives living in the household

**Table 3:** Background characteristics of stillbirths by verbal autopsy interview status

	Verbal autopsy interview				Chi-square
	incomplete		complete		test
	(n = 164)		(n = 1584)		p-value
	N	(%)	N	(%)	
Sex					
Male	108	(65.9)	892	(56.3)	
Female	55	(33.5)	678	(42.8)	
Missing	1	(0.6)	14	(0.9)	0.063
Maternal age					
14-19	2	(1.2)	34	(2.1)	
20-24	33	(20.1)	345	(21.8)	
25-29	44	(26.8)	434	(27.4)	
30-34	38	(23.2)	343	(21.7)	
35-39	31	(18.9)	286	(18.1)	
40-44	11	(6.7)	109	(6.9)	
45+	5	(3.0)	33	(2.1)	0.946
Maternal education					
None	86	(52.4)	755	(47.7)	
Primary incomplete	19	(11.6)	170	(10.7)	
Primary complete	28	(17.1)	308	(19.4)	
Secondary+	31	(18.9)	351	(22.2)	0.581
Interval between VA interview and end of pregnancy (months)					
0-11	24	(14.6)	289	(18.2)	
12-23	47	(28.7)	445	(28.1)	
24-35	52	(31.7)	530	(33.5)	
36-44	41	(25.0)	320	(20.2)	0.408
Plurality					
Singleton			1,469	(92.7)	
Multiple births			115	(7.3)	
Delivery place					
Home			1,253	(79.1)	
Facility			312	(19.7)	
On the way to a facility			9	(0.6)	
Other			10	(0.6)	
Delivery attendant					
None			175	(11.0)	
Family/Relatives			167	(10.5)	
Doctor			187	(11.8)	
Nurse/midwife			135	(8.5)	
Other health personnel*			24	(1.5)	
Traditional birth attendant †			878	(55.4)	
Quack			8	(0.5)	
Other			10	(0.6)	
Delivery mode					
Vaginal delivery without assistance			1,019	(64.3)	
Vaginal delivery with manual assistance			439	(27.7)	
Vaginal delivery with vacuum/forceps			20	(1.3)	
cesarean section after failure of instrument			56	(3.5)	
cesarean section			50	(3.2)	

Delivery characteristics are available for those who completed the verbal autopsy interview only.

*Including paramedics, family welfare visitors, health assistants, and family welfare assistants.

†Including both trained and untrained traditional birth attendants

Overall, 62% of stillbirths were classified as antepartum and 38% were intrapartum. However, in 131 of 965 antepartum stillbirths (13.6%), mothers reported paradoxically that the body was macerated yet that fetal movement had ceased prior to delivery, indicating potential reporting errors. In another 493 antepartum stillbirths, mothers reported cessation of fetal movement before onset of labor but no presence of maceration. The vast majority of the stillbirths occurred in term pregnancies, but, compared to intrapartum stillbirths, a larger proportion of antepartum stillbirths were in preterm pregnancies (Table 4). The distributions were comparable across the three years of the study (Table 2).

**Table 4:** Distribution of gestational age by timing of stillbirths: antepartum and intrapartum (n = 1554)*

gestational age (month)	anteparum		intrapartum	
	n	(%)	n	(%)
7	123	(12.7)	59	(10.0)
8	110	(11.4)	33	(5.6)
9	730	(75.6)	496	(84.2)
10	2	(0.2)	1	(0.2)

*chi-square test p-value < 0.000

Among antepartum stillbirths, based on the hierarchy of causes, about 29% had symptoms suggestive of maternal conditions, including infections (19%), hypertensive disorders (9%), or severe anemia (2%) (Table 5). Another 10% had maternal hemorrhage during pregnancy, and about 3% had congenital anomalies. A cause could not be assigned for 58% of the antepartum stillbirths using this methodology.

**Table 5:** Distributions of causes of stillbirths: antepartum and intrapartum (n = 1554)

Type	Cause	Non-hierarchical		Hierarchical	
		n	(%)	n	(%)
**Antepartum**	**Total**	**965**		**965**	(100.0)
	Congenital abnormality	25	(2.6)	25	(2.6)
	Maternal hemorrhage	101	(10.5)	100	(10.4)
	Maternal hypertensive disorder	90	(9.3)	82	(8.5)
	Maternal infections	225	(23.3)	179	(18.6)
	Maternal severe anemia	28	(2.9)	16	(1.7)
	Unexplained*	-	-	563	(58.3)
					
**Intrapartum**	**Total**	**589**		**589**	(100.0)
	Congenital abnormality	5	(0.8)	5	(0.9)
	Intrapartum hypoxia	322	(54.7)	319	(54.2)
	Preterm labour	67	(11.4)	43	(7.3)
	Maternal hypertensive disorder	53	(9.0)	27	(4.6)
	Maternal infections	104	(17.7)	32	(5.4)
	Unexplained*	-	-	163	(27.7)

* Residual category where no causes were assigned when a hierarchical approach was used.

The primary cause of about half of intrapartum stillbirths was intrapartum hypoxia, according to the hierarchy (Table 5), among which the most common cause was malpresentation (48%) (results not shown). Small proportions of intrapartum stillbirths were due to preterm labour (7.3%), maternal infections (5.4%), and maternal hypertensive disorders (4.6%). A cause could not be assigned in 28% of intrapartum stillbirths. Overall, 19% and 14% of the total stillbirths were due to obstetric complications and maternal infections, respectively. We further analyzed causes among singleton births only (n = 1444) (Table 6), and results were comparable with those based on all births.

**Table 6:** Distributions of causes of stillbirths among singleton births only: antepartum and intrapartum (n = 1444)

Type	Cause	Non-hierarchical		Hierarchical	
		n	(%)	n	(%)
**Antepartum**	**Total**	**912**		**912**	(100.0)
	Congenital abnormality	24	(2.6)	24	(2.6)
	Maternal hemorrhage	93	(10.2)	92	(10.1)
	Maternal hypertensive disorder	84	(9.2)	77	(8.4)
	Maternal infections	214	(23.5)	171	(18.8)
	Maternal severe anemia	28	(3.1)	16	(1.8)
	Unexplained*	-	-	532	(58.3)
					
**Intrapartum**	**Total**	**532**		**532**	(100.0)
	Congenital abnormality	5	(0.9)	5	(0.9)
	Intrapartum hypoxia	278	(52.3)	275	(51.7)
	Preterm labour	36	(6.8)	22	(4.1)
	Maternal hypertensive disorder	50	(9.4)	29	(5.5)
	Maternal infections	96	(18.1)	30	(5.6)
	Unexplained*	-	-	171	(32.1)

* Residual category where no causes were assigned when a hierarchical approach was used.

Sixty-two antepartum stillbirths and 117 intrapartum stillbirths met cases definitions of multiple causes, based on the non-hierarchical approach. Table 7 presents distribution of causes of stillbirths allowing for multiple causes based on the non-hierarchical approach, suggesting the primary-cause results were sensitive to changes in the hierarchy applied. For example, 23 antepartum stillbirths met the case definitions for maternal hemorrhage as well as maternal infections, and either cause was determined as the primary cause, depending on the order of the hierarchy.

**Table 7:** Overlapping multiple causes of stillbirths based on the non-hierarchical approach: antepartum and intrapartum (n = 1554)

Antepartum (n = 965)	Overlap with											
			
Cause	Congenital abnormality (n = 25)		Maternal hemorrhage (n = 101)		Maternal hypertensive disorder (n = 90)		Maternal infections (n = 225)		Maternal severe anemia (n = 28)		No overlap with other causes	
			
	n	(%)	n	(%)	n	(%)	n	(%)	n	(%)	n	(%)
Congenital abnormality (n = 25)			1	(4.0)	2	(8.0)	5	(20.0)	1	(4.0)	17	(68.0)
Maternal hemorrhage (n = 101)	1	(1.0)			6	(5.9)	23	(22.8)	2	(2.0)	73	(72.3)
Maternal hypertensive disorder (n = 90)	2	(2.2)	6	(6.7)			21	(23.3)	6	(6.7)	59	(65.6)
Maternal infections (n = 225)	5	(2.2)	23	(10.2)	21	(9.3)			6	(2.7)	175	(77.8)
Maternal severe anemia (n = 28)	1	(3.6)	2	(7.1)	6	(21.4)	6	(21.4)			16	(57.1)

**Intrapartum (n = 589)**	Overlap with											
			
Cause	Congenital abnormality (n = 5)		Intrapartum hypoxia (n = 322)		Preterm labour (n = 67)		Maternal hypertensive disorder (n = 53)		Maternal infections (n = 104)		No overlap with other causes	
			
	n	(%)	n	(%)	n	(%)	n	(%)	n	(%)	n	(%)

Congenital abnormality (n = 5)			3	(0.9)	1	(1.5)	0	(0.0)	2	(1.9)	0	(0.0)
Intrapartum hypoxia (n = 322)	3	(60.0)			23	(34.3)	23	(43.4)	61	(58.7)	220	(68.3)
Preterm labour (n = 67)	1	(20.0)	23	(7.1)			3	(5.7)	7	(6.7)	36	(53.7)
Maternal hypertensive disorder (n = 53)	0	(0.0)	23	(7.1)	3	(4.5)			10	(9.6)	21	(39.6)
Maternal infections (n = 104)	2	(40.0)	61	(18.9)	7	(10.4)	10	(18.9)			32	(30.8)

## Discussion

We used pregnancy history and verbal autopsy data to examine levels, timing, and causes of stillbirth in a rural population in Bangladesh, contributing to limited literature on stillbirth in developing countries. The estimated stillbirth rate was about 36 per 1000 total births. About 60% and 40% of these stillbirths were classified as antepartum and intrapartum, respectively. Maternal infections and hypertensive disorders, for which various interventions are available, contributed to about 21% of total stillbirths. About 50% of intrapartum stillbirths or 19% of all stillbirths were attributed to obstetric complications.

The estimated proportion of intrapartum stillbirths was roughly comparable with that reported in population-level studies conducted in Pakistan[18] and Ghana,[17] although the reported proportion ranged widely in other population-based studies, from 25% globally[5] to 86% in Nepal [29]. High proportions of intrapartum stillbirths were reported in studies where antepartum stillbirths were defined as "macerated",[6,29] whereas our study assigned antepartum stillbirth to those that were macerated or for which the mother reported that fetal movement stopped before the onset of labor. Our results are in agreement with previous studies in developing countries that have attributed high rates of stillbirth to the presence of a high burden of maternal conditions during pregnancy and obstetric complications during labor and delivery [17,18,30-32]. The cesarean section rate in our study population was relatively low (4.7%), suggesting there was an unmet need for emergency obstetric care which contributed to risk for intrapartum stillbirth. Direct comparison of our results on individual causes to previous studies is limited, however, due to differences in methodology. In addition to differences in questionnaires and definitions of causes across studies,[2,6,28,33] previous studies employed varying methods to assign causes such as physician review of the verbal autopsy data with[17] or without[18,30-32] structured hierarchical algorithms.

Despite limitations, verbal autopsy methodology is the only feasible method currently available for use in defining causes of stillbirths in settings where most births and deaths take place at home and a functional vital registration system does not exist [2,9,33,34]. In our study, well-trained field workers from the community administered the questionnaire, which was locally adapted to include culturally appropriate terms describing signs and symptoms. Further, high response rates in a population defined through a census and found to have high mortality provided complete verbal autopsy data on about 1600 stillbirths over the three-year study period.

Our findings, however, have a number of limitations. First, the most critical limitation is possible underestimation of stillbirths using pregnancy histories, as reported in a previous study [35]. In the home-care arm of our study, based on the prospective pregnancy and birth surveillance data collected by community health workers,[36,37] the stillbirth rate during 2004-2005 was estimated to be 50.4 per 1000 total births (562/11174), about 35% higher than our corresponding estimate based on retrospective pregnancy histories. While our neonatal mortality rate estimates were comparable between the prospective surveillance and retrospective pregnancy history data (data not shown), socio-cultural taboo might have discouraged reporting stillbirths in the retrospective survey of pregnancy histories [2]. Improvements in questionnaires and/or field administration of the questionnaire are needed to improve the ascertainment of stillbirth in pregnancy histories [7,18,21,29]. It is possible that women were more likely to omit antepartum stillbirths, especially those which occurred earlier during pregnancy, than intrapartum stillbirths,[17,35] potentially underestimating the burden of antepartum stillbirths. However, this needs further confirmation.

Second, we estimated timing of stillbirths based on maternal-report to two questions in the verbal autopsy questionnaire - 'macerated body' and 'cessation of fetal movement before labor'. However, accuracy of classification based on such reporting has not been validated,[9] and our results suggested potential issues in classification. Classification of stillbirths into antepartum and intrapartum would require examination of fetal remains for presence of signs indicating whether the fetus died more or less than 12 hours before delivery (antepartum and intrapartum stillbirths, respectively),[5] although there is potential misclassification using this definition [2,5,9]. However, socio-cultural factors may encourage rapid and secretive disposal of fetal remains in many cultures [2]. Mothers might not have seen the remains and/or family members who buried the remains might not have conveyed details of the remains to mothers, raising potential issues in validity of maternal report in questions regarding appearance of the remains. We speculate that macerated body might have been under-reported in our population.

Third, our case definitions used in both non-hierarchical and hierarchical expert algorithms, have not been validated. Use of the World Health Organization standardized verbal autopsy questionnaire along with the algorithms is likely to have low sensitivity for certain case definitions. For example, we only measured the lethal congenital abnormalities of the head and neural tube defects, and stillbirths due to other common congenital abnormalities, including chromosomal abnormalities and major cardiac defects were not included. Measurement of maternal infections is based on only two symptoms, whereas asymptomatic infections, including urinary and reproductive tract infections, are important causes of stillbirths. Hypertensive disorders and anemia may be underreported in this setting, since the definition consisted of convulsion or hypertension during pregnancy diagnosed by a health worker, while antenatal screening is not universal and is often of poor quality in the study area. A substantial proportion of stillbirths (49% of total) was unexplained in our study, but this level is comparable to previous studies using verbal autopsy data [17,18]. Even in settings with adequate obstetric care, stillbirth often remains an event without clear cause [38].

In addition, the high degree of overlap between causes in our data suggests that the results are sensitive to changes in the hierarchy applied. There is relatively little experience in the use of verbal autopsy and hierarchical algorithms to assign the primary cause for stillbirth,[33] whereas there is ample experience of using verbal autopsy to ascertain causes of neonatal deaths [10-14,39] and growing agreement exists regarding use of hierarchical algorithms to assign the primary causes of neonatal deaths [15,40]. Only one study, using data from the Obaapa trial conducted in rural central Ghana, validated hierarchical algorithms to assign primary cause of stillbirths based on verbal autopsy data. The study reported high sensitivity and specificity of their algorithm in identifying intrapartum stillbirths due to obstetric complications but lower validity for other causes compared to physician review [16]. Also, sensitivity and specificity were generally lower for identifying causes of stillbirths compared to causes of neonatal deaths [16].

Finally, the mean recall period of 25 months in our study is longer than that generally reported [28]. However, the relationship between reporting errors and recall periods has been studied mainly using verbal autopsy data for adult deaths, where the respondent's recall may not be as accurate as maternal recall of symptoms and signs preceding stillbirths or child deaths [28].

Despite these limitations in measurements, our findings on stillbirth burden and etiology have important programmatic implications for preventing stillbirths in rural Bangladesh and in similar settings. About half of the intrapartum stillbirths, or about 19% of total stillbirths, resulted from obstetric complications. Maternal conditions, including hypertensive disorders, infections, and anemia, contributed to 29% of antepartum stillbirths and 11% of intrapartum stillbirths. In particular, maternal infections contributed to 14% of total stillbirths in our population, suggesting the need to address this complication at the community level. A recent population-based study on neonatal bacteremia also suggested the potential significance of preventing vertical transmission of maternal infections to reduce neonatal infections in communities [27].

Preventive and curative interventions to address maternal complications are available, but have not been scaled up effectively in many low-resource settings [41,42]. Maternal infections during pregnancy include a variety of conditions such as urinary and reproductive tract infections, sexually transmitted infections, malaria, and infection following or causing premature rupture of membranes. Prophylactic antibiotic treatment may reduce the risk of maternal infections and premature rupture of the membranes,[43,44] although evidence on the reduction in perinatal mortality is inconclusive [42]. Routine screening and treatment of syphilis is an effective intervention to reduce perinatal mortality, although the disease burden is thought to be low in our study population [41,42]. Prophylactic treatment and insecticide-treated bednets are also effective interventions to reduce perinatal mortality in malaria endemic areas [41,42,45-47]. Hypertensive disorders during pregnancy include a wide range of conditions,[48,49] but pregnancy-induced hypertension and preeclampsia may be effectively prevented with calcium supplementation during pregnancy [41,42,50]. Finally, nutritional interventions during pregnancy to address both macro- and micronutrient deficiencies have the potential to address important contributing factors for stillbirths,[41] although further evidence of their effectiveness in reducing stillbirths is needed [41,51].

In order to reduce intrapartum stillbirths, strategies to improve access to skilled childbirth care and management of obstetric complications during labor and delivery are essential [41,52,53]. Such interventions include improving birth preparedness[54,55] and increasing access to skilled birth attendance at home[56-59] and high quality emergency obstetric care, in particular including Caesarean section and access to essential drugs [53,60,61]. To improve access to these interventions, strategies such as community mobilization, financial incentive schemes, and community referral/transport systems have been suggested to be promising [62]. Magnesium sulfate can effectively prevent eclamptic seizures during labor, although its impact on perinatal mortality has not been demonstrated [52]. Finally, maintaining quality of care during labor is essential as improper use of uterotonics is associated with increased risk of stillbirth [63].

## Conclusions

We identified a high burden of stillbirths in rural Bangladesh. Nearly two-thirds of stillbirths occurred in the antepartum period. The etiology of stillbirths implies that optimal management of maternal complications during pregnancy and obstetric complications during labor are required to avert stillbirths in this population. Proven interventions are available with the potential to substantially reduce stillbirth rates; these should be scaled in the context of maternal and child health programs. The causes of about half of the stillbirths could not be ascertained. Methodologic improvement in measurement of stillbirths, in particular causes of stillbirths, is critical to better understand and avert stillbirths in low-resource settings.

## Competing interests

The authors declare that they have no competing interests.

## Authors' contributions

AHB, SEA, GLD, and REB participated in the research and intervention design and implementation, data collection, data analysis and manuscript writing. YC participated in data analysis and manuscript writing. EKW participated in manuscript writing. IM participated in the research and intervention design and implementation and data collection. All authors contributed to preparation of the manuscript.

## Pre-publication history

The pre-publication history for this paper can be accessed here:

http://www.biomedcentral.com/1471-2393/11/25/prepub
